# Importance of Behavioral Therapy in Patients Hospitalized for Post-Traumatic Stress Disorder (PTSD) with Opioid Use Disorder

**DOI:** 10.3390/bs8080073

**Published:** 2018-08-12

**Authors:** Rikinkumar S. Patel, Geetha Manikkara, Priya Patel, Jupi Talukdar, Zeeshan Mansuri

**Affiliations:** 1 Department of Psychiatry, Griffin Memorial Hospital, Norman, OK 73071, USA; 2 Department of Psychiatry, Texas Tech University Health Science Center, Midland, TX 79701, USA; geethamanikkara@gmail.com (G.M.); zeeshan.mansuri@ttuhsc.edu (Z.M.); 3 School of Medicine, Windsor University, Monee, IL 60449, USA; priya.patel1810@gmail.com; 4 Gauhati Medical College and Hospital, Bhangagarh, Guwahati, Assam 781032, India; jupi1589@gmail.com

**Keywords:** post-traumatic stress disorder, PTSD, opioid use, behavioral therapy, inpatient psychiatry

## Abstract

**Objective**: To analyze differences in demographic pattern and hospitalization outcomes in post-traumatic stress disorder (PTSD) with opioid use disorder (OUD) patients managed with versus without behavioral therapy (BT). **Methods**: We conducted case-control study using Nationwide Inpatient Sample and identified PTSD and OUD using ICD–9–CM codes. Linear regression model was used to evaluate impact of BT on inpatient stay and cost. **Results**: We analyzed 1531 inpatient admissions and 786 patients received BT. Females had higher odds of receiving BT during inpatient management for PTSD with OUD (OR 1.210; 95% CI 1.020–1.436). About 63.1% patients receiving BT were benefited by Medicaid. Patients receiving BT had an increase in hospital stay by 1.27 days (P = 0.085) and hospitalization cost by $4734 (P = 0.018). There were no transfers to short term hospitals and lower transfers to skilled nursing facility (3.8% vs. 10.1%) in patients receiving BT. **Conclusion**: This study aims to reinforce combination management with psychotropic medications and BT in PTSD patients with comorbid OUD during hospitalization as it significantly decreases adverse disposition of the patient and thereby improves the quality of life post-treatment.

## 1. Introduction

Post-Traumatic Stress Disorder (PTSD) represents a syndrome of symptoms experienced by some individuals after a traumatic event like wars, physical abuse, sexual abuse, accidents, natural disasters and terrorist attacks. The symptoms typically start within three months of the event and should last more than a month [1]. Under Diagnostic and Statistical Manual of Mental Disorders (DSM–5) [2], for those six years of age and above, PTSD includes four clusters of symptoms– re-experiencing the event in form of traumatic nightmares, dissociative reactions or prolonged psychological distress, alterations in arousal like aggression, recklessness or self-destructive behavior, sleep disturbances or hypervigilance, avoidance of distressing memories or thoughts of the event, and lastly negative alterations in cognition and mood.

Opioids exert their action on a neurological level and alter the chemistry of the brain to promote euphoria and extreme relaxation, which can lead to distraction from discomforting symptoms [3]. Several studies have indicated that there is a strong relationship between PTSD and substance abuse [4,5,6,7,8]. A possible rationale for opioid abuse is the relief from anxiety evoked from re-experiencing symptoms where the affected individual might feel the same amount of fear and helplessness experienced during the actual trauma. Very often, the individuals experiencing PTSD symptoms abusing opioids run into withdrawal because of sudden unavailability of the drug. The withdrawal symptoms are similar to the category of ‘arousal’ symptoms of PTSD which presents as severe irritability, inability to sleep or concentrate and being easily startled [9].

PTSD has a high co-occurrence with OUD. Earlier literature found a prevalence rate of 6.6% for comorbid PTSD and prescription opioid dependence among civilians. It has been found that patients with OUD have a 42% increased likelihood of having PTSD [3]. The coexistence of PTSD with OUD is being studied to some extent. It is also being understood that the outcome of PTSD patients who also abuse opioids is worse in terms of prognosis [10]. Studies show that if left untreated, patients with comorbid PTSD with OUD have a decreased quality of life, higher likelihood of continued substance use and overdose, worse occupational functioning [11]. Therefore, it is imperative to treat this comorbidity effectively.

The preferred treatment is an integrated therapy consisting of both behavioral therapy (BT) and psychotropic medications. In a study by Saunders et al. it was found that integrated CBT in combination of psychotropic medications may demonstrate an improvement in substance use outcomes for patients who also have PTSD as compared to psychosocial treatment alone [11]. Previous research has highlighted the importance of both psychotropic medications and BT in improving outcomes in the treatment of PTSD with OUD [11,12]. There was significantly positive outcome with combined therapy in remission and post treatment outcomes [13,14,15,16,17].

The primary objectives of this study are to analyze and discern the variance in the demographic pattern, inpatient length of stay and hospitalization cost, and disposition in patients managed with BT during hospitalization versus without BT. To effectively assess the patient care of PTSD with OUD, the results obtained from this study will allow for strategic developments of clinical care models to screen and treat both conditions, and improve utilization of BT in during hospitalization.

## 2. Methods 

### 2.1. Source of Data

We collected data from the National Inpatient Sample (NIS) of Healthcare Cost and Utilization Project [18]. The database processes and stores information on inpatients admissions and discharges from approximately 1050 hospitals across the United States of America. Per year, it records cross-sectional data on eight million inpatient admissions and discharges and reflects a 20% stratified sample of all non-federal hospitals. It is the largest all payers’ dataset accessible at present. The information on each admission comprises one primary discharge diagnosis and up to 25 secondary diagnoses. The dataset does not include information on long-term care and rehabilitation facilities utilized. As the data is unweighted, the weighted estimate of the total discharge number of the US population results when the discharge weight is applied to the unweighted data. The NIS is sponsored by the Agency for Healthcare Research and Quality (AHRQ) [18] and does not require the approval from the Institutional Review Board (IRB) as the data is de-identified.

### 2.2. Case and Control Selection

All the patients are between 18–65 years’ age. The cases and controls were identified by the International Classification of Diseases, 9th Revision, and Clinical Modification (ICD–9–CM) diagnosis codes, with a primary diagnosis of PTSD and secondary diagnosis of OUD, and primary treatment of psychotropic medications. Correspondingly, based on the ICD–9–CM procedure codes, the cases were managed by secondary treatment of BT whereas the control group was managed without BT. Controls were matched with the cases for age, with the group indicator based on behavioral therapy (yes or no) and case ID using case-control matching on SPSS version 23.0 [19].

In HCUP databases, more than 14,000 ICD-9-CM diagnosis codes and 3900 procedure codes had been mentioned [18]. PTSD was identified using ICD–9–CM diagnosis code 309.81; and OUD was identified using diagnosis codes 304.00, 304.01, 304.02, 305.50, 305.51 and 305.52. Psychiatric drug treatment and BT was identified using ICD–9–CM procedure code 94.25 and 94.33 respectively. Patients receiving other psychotherapies during hospitalization were identified by ICD–9–CM procedure codes 94.39 (individual therapy) and 94.44 (group therapy). 

### 2.3. Variables of Interest

To measure the differences in hospitalization outcomes in patients managed with CBT versus without CBT, the outcome variables of interest included the severity of illness and disposition of the patient. The All Patient Refined DRGs (APR-DRGs) are allocated using 3M Health Information Systems software. This severity measure includes the APR-DRG, the severity of illness subclass within each base APR-DRG and is measured at the time of inpatient admission [20]. We calculated the length of inpatient stay as the number of nights the patient remained in the hospital for a particular primary diagnosis. The length of inpatient stay in this analysis was all-cause. Inpatient charges during hospitalization do not include professional fees and non-covered charges [20]. 

### 2.4. Approaches

This study presents a retrospective analysis of the demographics and hospital outcomes for patients with BT versus without BT by applying data from the NIS [18]. We summarized the results with the help of descriptive statistics. We expressed categorical data using Pearson’s Chi-square test and continuous data by using Independent Sample T-test. The categorical variables were denoted by counts and percentages. General linear multiple regression analysis was used to estimate the association of BT with the length of stay and total hospital charges. A multinomial logistic regression model was used to measure the likelihood of associations using Odds Ratio (OR) and 95% Confidence Interval (95% CI) between cases and controls (reference category) in terms of demographics and disposition. Discharge weights were applied in all regression models to obtain nationally representative inpatient data. A P value < 0.05 was chosen to be the reference value to denote statistical significance. SPSS version 23.0 was used to do all the statistical analysis in this study [19].

## 3. Results

### 3.1. Demographic Characteristics 

A total of 1531 patients were included in the study population and 786 patients received BT as secondary management during hospitalization. The mean age of the study population was 41.77 years and about 53.6% patients were young adults (18–35 years). About 1000 patients (65.4% of total) admitted for inpatient management of PTSD with OUD were males, and BT was utilized in 63.1% males. Yet, females had higher odds of receiving BT during inpatient management for PTSD with OUD (OR 1.210; 95% CI 1.020–1.436; P = 0.029) compared to patients without BT management. More than half of the study population were Caucasians (56.1%) and BT was utilized in 51% Caucasians followed by African Americans (22.3%). received BT as secary management About two-fifth of all the patients were covered by Medicaid, and so 63.1% patients receiving BT were benefited by Medicaid. There were two-fold higher likelihood patients self-paying for the hospital cost to receive BT (OR 2.250; 95% CI 1.329–3.810; P = 0.003). The demographic distribution of the study population based upon CBT management is shown in Table 1. 

### 3.2. Differences in Inpatient Outcomes

Majority of the patients admitted for PTSD with comorbid OUD had moderate morbidity at the time of admission. When 5.6% of the total patients had severe morbidity, then only 3.2% received BT. Patients managed with BT had higher utilization of individual therapy and group therapy (57.9% and 75.8%; respectively) compared to those without BT management. 

The mean length of inpatients stays and cost per admission was higher in patients receiving BT compared to those without BT management as shown in Table 2. Patients receiving BT had an increase in overall hospital stay by 1.27 days per admission (95% CI −0.18 to 2.72; P = 0.085) and the total hospitalization cost by $4734 (95% CI 811.53 to 8656.26; P = 0.018). Another factor that increases the stay (3.2 days; 95% CI 1.44 to 4.88; P < 0.001) and cost ($4538; 95% CI −135.54 to 9211.67; P = 0.047) during hospitalization was public health coverage (Medicare and Medicaid).

A higher proportion of patients with PTSD and OUD receiving BT were discharged home (94.3% vs. 83.2%) and so had higher likelihood of routine disposition compared to those without BT management (OR 1.194; 95% CI 1.073–1.329; P = 0.001). There were no transfers to short term hospitals and lower transfers to skilled nursing facility or intermediate nursing facility (3.8% vs. 10.1%) in patients receiving BT during inpatient hospitalization.

## 4. Discussion

This retrospective study of population-based hospital data from patients admitted for PTSD with comorbid OUD and receiving psychotropic medications as primary treatment were further differentiated on the basis of utilization of BT in order to study the demographic predictors of BT and difference in the hospitalization outcomes. In our study we analyzed 1531 inpatient admissions for PTSD with OUD. BT was utilized in 786 patients as an integral part of psychotherapeutic management during hospitalization. 

Our study revealed that a female had higher likelihood of being managed by BT for PTSD with comorbid OUD during hospitalization. BT was utilized more by the Caucasians, but when we relate the proportion of African Americans constituting the total sample and those who received BT, then African Americans were more likely to be managed with BT. About half of the patients were covered by Medicaid making it the most prevalent primary payer. 

The coexistence of PTSD with OUD can be possibly attributed to an urge to ‘escape’ re-experiencing a traumatic event by mediating neurotransmitters with drugs like opioid which have a calming effect [12]. A previous study by Saunders et al. found that integrated BT with MAT may demonstrate an improvement in substance use outcomes for patients with PTSD and comorbid OUD [11]. Our study supports this, as higher proportion of patients who were treated with BT also received group and individual psychotherapy during hospitalization and were discharged routinely compared to those without receiving BT. But, we also noticed that BT was used less in patients with severe morbidity compared to highest utilization in patients with moderate loss of body function. 

Mills et al., in their study reported no significant difference in the cost of treatment over a twelve months’ period in patients with PTSD and comorbid OUD [21]. But in our study we found that patients with BT increased the total length of hospitalization by 1.3 days and total hospitalization charges by $4734. There were lower proportion of adverse disposition in patients managed with BT compared to those without BT.

The biggest strengths of this study are the uniform collection of data through ICD-9-CM codes as well as the national representation provided by NIS. To our knowledge, this is the first study, to report the demographic and hospitalization differences in PTSD patients with OUD receiving BT versus without BT. Another strength is the reliability of the data as it is coded independently of the individual practitioner so there is minimal reporting bias.

However, our study does have some limitations. Accurate clinical associations cannot be made, owing to the fact that NIS is an administrative database and therefore lacks the required patient level data. The sample in this study is non-BT versus BT is based upon the detection of BT as secondary treatment using ICD-9-CM procedure code during the entire hospitalization course. The ICD-9-CM codes are used depends largely on external factors such as insurance and billing, and absence of BT treatment codes do not necessarily reflect absence of administration of BT-informed techniques. Also, being a retrospective study, it is subject to selection bias, and due to the moderate sensitivity of diagnostic codes for PTSD, OUD and BT this bias may be further increased. Also, re-hospitalizations which is a factor into total inpatient burden are not accounted for, given the nature of the database. Despite these potential limitations, the NIS database provides an unparalleled population-based perspective on disease associations to help demonstrate the importance of BT for management of PTSD with OUD patients thereby providing a rationale for further studies on this approach.

## 5. Conclusions 

This study aims to reinforce combination management with psychotropic medications and behavioral therapy in PTSD patients with comorbid opioid use disorder during hospitalization. Increase use of behavioral therapyin managing patients with severe morbidity should be encouraged. Though, behavioral therapy increases the hospitalization stay and cost but it significantly decreases adverse disposition of the patient. Given the benefits behavioral therapy leading to an overall improvement in outcomes, this approach is worthy of further investigation, in order to be considered as definitive treatment for PTSD patients with opioid use disorder to improve the quality of life.

## Figures and Tables

**Table 1:** Demographic distribution of patients by BT.

Characteristics	BT (−)		BT (+)		Total		P
	N	%	N	%	N	%	
**Age at time of admission**							
18–35 years	410	55.0	411	52.3	821	53.6	0.282
36–65 years	335	45.0	375	47.7	710	46.4	
**Gender**							
Male	505	67.8	495	63.1	1000	65.4	0.052
Female	240	32.2	290	36.9	530	34.6	
**Race**							
Caucasian	455	61.5	400	51.0	855	56.1	<0.001
African American	145	19.6	175	22.3	320	21.0	
Hispanic	95	12.8	95	12.1	190	12.5	
Others	45	6.1	115	14.6	160	10.5	
**Primary Payer/Insurance**							
Medicare	135	18.1	120	15.3	255	16.7	<0.001
Medicaid	420	56.4	495	63.1	915	59.8	
Private	115	15.4	95	12.1	210	13.7	
Self-pay	20	2.7	45	5.7	65	4.2	
Other	55	7.4	30	3.8	85	5.6	
**Median household income**							
0–25th percentile	170	28.1	160	27.8	330	28.0	0.176
26th–50th percentile	110	18.2	110	19.1	220	18.6	
51th–75th percentile	145	24.0	110	19.1	255	21.6	
76th–100th percentile	180	29.8	195	33.9	375	31.8	

Significant P ≤ 0.05 at 95% Confidence Interval; P values were obtained between BT (−) and BT (+) by using the Pearson Chi-Square (χ2) test. PTSD: Post-traumatic Stress Disorder; OUD: Opioid use Disorder; BT: Behavioral Therapy.

**Table 2:** Inpatient Outcomes distribution of patients by BT.

Characteristics	BT (−)		BT (+)		Total		P
	N	%	N	%	N	%	
**Severity of illness at admission**							
Minor	165	22.1	255	32.4	420	27.4	<0.001
Moderate	520	69.8	506	64.4	1026	67.0	
Major	60	8.1	25	3.2	85	5.6	
**Inpatient length of stay and cost per admission**							
Mean stay	9.68 days		11.14 days		10.43 days		0.048
Mean cost	$30,556		$35,762		$33,169		0.009
**Other psychotherapies**							
Group therapy	0	0	596	75.8	596	38.09	<0.001
Individual therapy	0	0	455	57.9	455	29.7	<0.001
**Disposition or discharge status**							
Routine	620	83.2	740	94.3	1360	88.9	<0.001
Short term hospital	15	2.0	0	0	15	1.0	
SNF/INF	75	10.1	30	3.8	105	6.9	
HHC	10	1.3	0	0	10	0.7	
AMA	25	3.4	15	1.9	40	2.6	

Significant P ≤ 0.05 at 95% Confidence Interval; P values were obtained between BT (−) and BT (+) by using the Pearson Chi-Square (χ2) test. PTSD: Post-traumatic Stress Disorder; OUD: Opioid use Disorder; BT: Behavioral Therapy; SNF: Skilled Nursing Facility; INF: Intermediate Nursing Facility; HHC: home health care; AMA: against medical advice.
